# Checkpoint-inhibitor induced Polyserositis with Edema

**DOI:** 10.1007/s00262-022-03211-7

**Published:** 2022-05-16

**Authors:** Sarah Zierold, Larissa Semra Akcetin, Eva Gresser, Anna Marie Maier, Alexander König, Rafaela Kramer, Sebastian Theurich, Dirk Tomsitz, Michael Erdmann, Lars E. French, Martina Rudelius, Lucie Heinzerling

**Affiliations:** 1 Department of Dermatology and Allergy, University Hospital, LMU Munich, Frauenlobstraße 9-11, 80337 Munich, Germany; 2 https://www.serio-registry.org/; 3 Department of Radiology, University Hospital, LMU Munich, Munich, Germany; 4 Department of Obstetrics and Gynecology, University Hospital, LMU Munich, Munich, Germany; 5 Department of Dermatology, Friedrich-Alexander-Universität Erlangen-Nürnberg (FAU), Universitätsklinikum Erlangen, Erlangen, Germany; 6 Deutsches Zentrum Immuntherapie (DZI), Erlangen, Germany; 7 Comprehensive Cancer Center Erlangen-European Metropolitan Area of Nürnberg (CCC ER-EMN), Erlangen, Germany; 8 Department of Medicine III, University Hospital, LMU Munich, Munich, Germany; 9 German Cancer Consortium (DKTK), Partner site Munich, Munich, Germany; 10 German Cancer Research Center (DKFZ), Heidelberg, Germany; 11 Dr. Philip Frost, Department of Dermatology and Cutaneous Surgery, University of Miami Miller School of Medicine, Miami, FL USA; 12 Institute of Pathology, Ludwig-Maximilians-Universität München, Munich, Germany

**Keywords:** Autoimmunity, Anti-PD1-antibody, Side effects, Serositis, Pericardial effusion, Pleural effusion

## Abstract

**Background:**

As immune checkpoint inhibitors (ICI) are increasingly being used due to effectiveness in various tumor entities, rare side effects occur more frequently. Pericardial effusion has been reported in patients with advanced non-small cell lung cancer (NSCLC) after or under treatment with immune checkpoint inhibitors. However, knowledge about serositis and edemas induced by checkpoint inhibitors in other tumor entities is scarce.

**Methods and results:**

Four cases with sudden onset of checkpoint inhibitor induced serositis (irSerositis) are presented including one patient with metastatic cervical cancer, two with metastatic melanoma and one with non-small cell lung cancer (NSCLC). In all cases treatment with steroids was successful in the beginning, but did not lead to complete recovery of the patients. All patients required multiple punctures. Three of the patients presented with additional peripheral edema; in one patient only the lower extremities were affected, whereas the entire body, even face and eyelids were involved in the other patients. In all patients serositis was accompanied by other immune-related adverse events (irAEs).

**Conclusion:**

ICI-induced serositis and effusions are complex to diagnose and treat and might be underdiagnosed. For differentiation from malignant serositis pathology of the punctured fluid can be helpful (lymphocytes vs. malignant cells). Identifying irSerositis as early as possible is essential since steroids can improve symptoms.

## Background

Immune checkpoint inhibitors (ICI) are increasingly important due to their effectiveness in various tumor entities. However, checkpoint inhibitor therapy is associated with various immune-mediated side effects, which can affect every organ system and potentially be fatal [1]. The most frequent irAEs are not necessarily the most difficult ones to treat. While irColitis is frequent it can mostly be well managed while cardiovascular toxicities are rare, but associated with a high mortality rate [2]. Multiple manifestations of immune-related cardiac symptoms have been described, including autoimmune myocarditis, cardiomyopathy and cardiac arrest [3]. Besides myocarditis, pericarditis seems to be the most common manifestation of ICI induced cardiotoxicity [4]. A recently published pharmacovigilance analysis of the FDA found 16,862 reports of pericardial disorders related to ICI between 2011 and 2020, raising the possibility that it occurs more frequently than initially thought [5].

Autoimmune serositis (irSerositis) represents an especially difficult to treat complex of symptoms beyond pericardial effusion, with non-infectious inflammation of pericardium, pleura, and peritoneum. Currently, mainly case reports of pericardial effusion under nivolumab in patients with advanced non-small cell lung cancer (NSCLC) are available [6–8]. Some of them are reported to require pericardiocentesis due to cardiac tamponade [9]. There are also reports of recurrent pleural effusions under nivolumab [9]. The significance of pleural effusion as a prognostic factor has been controversial. While one report indicates it is a negative prognostic factor in patients with NSCLC [10], another report documents that the detection of a high CD4/CD8 quotient in the pleural fluid might predict better outcome in patients with advanced lung cancer receiving ICIs [11].

Taken together data on clinical presentation, response to therapy and outcome of immune checkpoint induced serositis is lacking especially in tumor entities other than metastatic lung cancer.

### Case 1

**Fig. 1:**
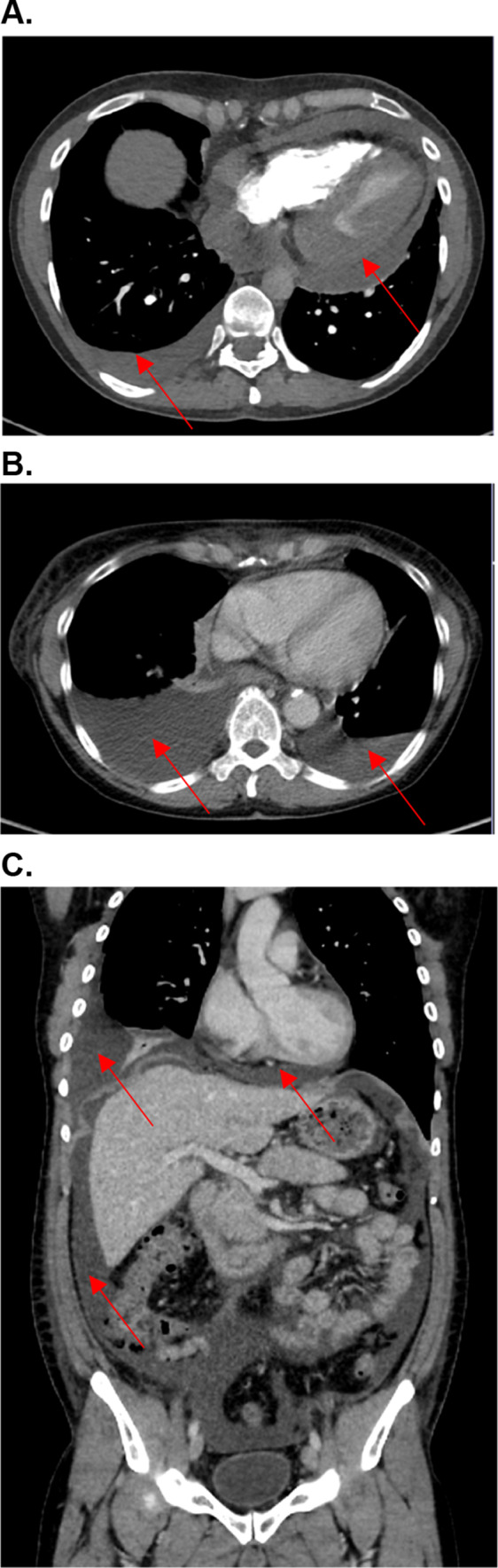
**A** Case 1. CT angiography of the arteria pulmonalis. Pulmonary arterial contrast phase to rule out pulmonary embolism. Circular pericardial effusion with rim up to about 2 cm and low density enhancement up to about 20–30 HU (Hounsfield units). Furthermore, serous pleural effusion on the right of approximately 2 cm. **B** Case 3. PET/CT with 232 MBq F-18 FDG, venous phase. Serous pleural effusions (approximately 15 Hounsfield units). Right > left up to 6.2 cm. **C** Case 4. Marked four-quadrant ascites (approximately 5–10 Hounsfield units (HU)). Basal serous pericardial effusion approximately 1.5 cm (up to 10–15 HU) as well as serous pleural effusion on the right

A 41-year-old woman was diagnosed with metastatic carcinoma of the cervix during pregnancy (14th week; initial tumor stage pT1b, pN0). Six months after giving birth, diagnostic laparoscopy was performed to complete the diagnostic and already showed peritoneal metastases, lymph node metastases and a local recurrence of the tumor. Chemotherapy with paclitaxel, cisplatin and bevacizumab was initiated and the subsequent staging showed tumor response. Due to neurological symptoms a MRI of the head was performed and cerebral metastases were detected. An immunotherapy with ipilimumab (3 mg/kg body weight (bw)) and nivolumab (1 mg/kg bw) was initiated for four cycles before the patient developed an immune-related hepatitis (CTCAE grade 4). Systemic steroids and discontinuation of the ICI therapy led to a normalization of liver enzymes. Due to nodal progress in the therapy-free interval monotherapy with nivolumab under close monitoring of the liver enzymes was reinitiated. Under nivolumab the liver enzymes remained controlled, but after four infusions, the patient presented in the emergency room with massive edema of the legs, swelling of her face and eye lids, progressive shortness of breath due to pleural effusion and a pericardial effusion with beginning hemodynamic relevance (Fig. 1A). Blood results showed acute renal failure with an immune-related nephritis (irNephritis). Diagnostic investigations did not reveal any cause which could have explained the sudden clinical symptoms. Pleura as well as pericardial puncture was conducted due to progressive dyspnea. Interestingly, pericardial fluid analysis identified malignant cells, whereas the pleural fluid showed lots of lymphocytes as a sign of acute inflammation without any malignant cells. In association with the irNephritis the serositis was also assumed to be immune-related and consequently high dose systemic steroid therapy was initiated with prednisone 1 mg/kg body weight. Unfortunately, this did not lead to a significant clinical improvement. The steroid dose was increased to 2 mg/kg body weight, which finally improved the kidney function, but only resulted in a modest reduction of the edema. According to progression of the previously known pleural and pericardial effusion, another pleural and pericardial puncture was performed, interestingly now detecting malignant cells also in the pleural fluid. The clinical condition of the patient rapidly deteriorated and she died due to multiple organ failure. The recurrent pericardial and pleural effusion as well as the edemas were thought to be multifactorial; due to tumor progression, and also as part of an immune-related reaction.

### Case 2

A 73-year-old male patient with melanoma of unknown primary and cerebral metastases with previously known dermatomyositis was started on monotherapy with pembrolizumab rather than combined immunotherapy with ipilimumab and nivolumab to decrease the possible risk of an exacerbation of the autoimmune disease. The dermatomyositis was treated with high-dose immunoglobulins before and remained stable under ICI therapy. One year after the initiation of pembrolizumab (15 cycles) the patient presented to the emergency room with diarrhea, massive edema of both legs and swelling of the scrotum. Furthermore, he complained about progressive dyspnea and a huge pleural effusion could be confirmed in a chest x-ray. Pleural fluid analysis identified abundant lymphocytes but no malignant cells, thus leading to the diagnosis of an immune-related pleuritis after exclusion of alternative causes. Abdominal sonography showed ascites in perihepatic and perisplenic areas, with low volumes that did not require puncture. A therapy with prednisone (0.5 mg/kg bw) was initiated, and initially led to a moderate reduction of the edema as well as the pleural effusion.

Similar to case one, the patient presented several times due to recurrent pleural effusion with subsequent necessary pleural punctures after he was discharged from the hospital. Steroids induced a decent improvement, but never led to complete recovery, even in higher dosage (1 mg/kg bw). To relieve the pleural effusion not only temporarily, a pleurodesis was performed. In every pleural fluid analysis merely lymphocytes, but no malignant cells were identified.

### Case 3

A 68-year-old woman was diagnosed with NSCLC stage IV with cerebral, pulmonal and nodal metastases. After radiotherapy of cerebral metastases and six cycles of pemetrexed/carboplatin, staging showed stable disease, and monotherapy with pemetrexed was continued (10 cycles). Due to a progress under pemetrexed, therapy with nivolumab was initiated. Fortunately, a PET/CT as well as a MRI of the brain showed stable disease. After 15 cycles of nivolumab the patient complained about progressive exertional dyspnea. Diagnostic work-up revealed pleural effusions on both sides requiring multiple pleural punctures (Fig. 1B). Each pathological investigation identified a plasmo-lymphocytic infiltrate in the pleural fluid, whereas no malignant cells were detected. Furthermore, imaging detected ascites in all four quadrants of the abdomen, although no paracentesis was necessary due to size consistency. Corticosteroid therapy was initiated with 1 mg/kg body weight, which initially improved symptoms. Thus, corticosteroids were tapered and immunotherapy with nivolumab was continued for up to 28 cycles, under which the NSCLC remained stable. In addition to recurrent pleural effusions, the patient developed an immune-related thyreoiditis with subsequent hypothyroidism. Due to persistent pleural effusions therapy with nivolumab had to be discontinued. Since then staging showed stable disease. Pleural effusions have been stable since the discontinuation of the ICI therapy 15 months ago and no longer required puncturing.

### Case 4

**Fig. 2:**
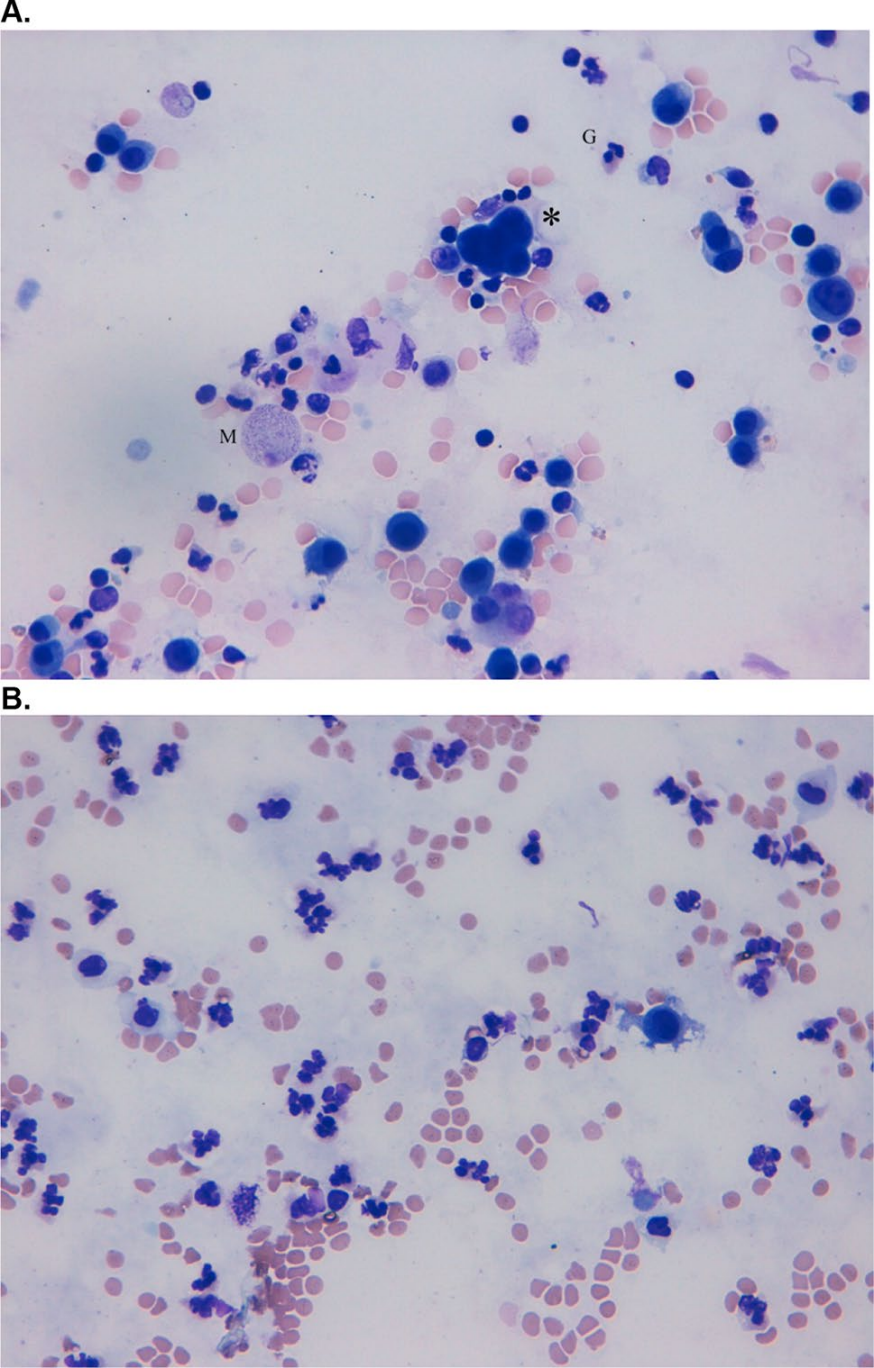
**A** Giemsa staining of malignant pleural effusion. Carcinoma cells (*) are intermingled with granulocytes (G) and macrophages (M). **B** Giemsa staining of ICI-induced serous pleural effusion. Numerous segmented granulocytes and few serosa cells are displayed

A 32-year-old patient with metastatic melanoma (Stage IIIC AJCC) received adjuvant therapy with pembrolizumab. Nine weeks after initiation of the immunotherapy he presented with nervousness and inner restlessness. Laboratory tests showed an inadequately increased fT4 in the context of an immune-mediated thyreoiditis, which turned into a hypothyroidism four weeks later, requiring L-thyroxin substitution. Three weeks later he presented to the emergency room with swelling of his face, painless scrotum swelling as well as a weight gain of 7 kg within two weeks and fatigue. Further diagnostic showed ascites as well as pleural effusions (Fig. 2C). Laboratory results demonstrated a thrombocytopenia (16.000 G/l), without any sings of bleeding. Due to the assumption of an immune-mediated polyserositis as well as immune-related thrombocytopenia, a therapy with prednisolone was initiated (1 mg/kg bw) for four weeks. The edema slowly regressed and a weight loss of 3 kg was achieved. ICI therapy was interrupted after four cycles and was not reinitiated. One week after discontinuation of the therapy with prednisolone the patient presented to the emergency room with dyspnea. Pleural puncture was performed and pathological investigation of the pleural fluid detected lymphocytes in the absence of malignant cells. Furthermore, peritoneal fluid detected no signs of malignancy. Echocardiography showed a minimal, hemodynamically irrelevant pericardial effusion, which remained stable in following investigations. Due to irPolyserositis therapy with prednisolone (1 mg/kg bw) was reinitiated and tapered over three months. Under the therapy with prednisolone bilateral pleural effusions remained stable even after a dose reduction and also after discontinuation. A singular brain metastasis was detected during a staging one year and a half year after discontinuation of the immunotherapy with pembrolizumab. Due to severe immune-mediated side effects under pembrolizumab and a BRAF-V600E mutation, a targeted therapy was initiated. An overview of patient characteristics, clinical manifestation an outcome is given in Table 1.

**Table 1:** Patient characteristics, clinical manifestation and outcome

Case Nr	Gender	Age	Tumor entity	Tumor therapy	Clinical manifestation	Onset after initiation of immunotherapy (weeks)	Response to steroids	Recurrence (Y/N)	Response to tumor therapy	Additional irAE
1	female	42	carcinoma of the cervix	Nivolumab	pleural and pericardial effusion, edema of the legs, swelling of face and eyelids	8	modest improvement	Y	PD	irHepatitis (grade 4), irNephritis (grade 2)
2	male	73	melanoma of unknown primary	Pembrolizumab	pleural effusion, ascites, edema of the legs	52	modest improvement	Y	PD	irColitis (grade 2)
3	female	69	NSCLC	Nivolumab	Pleural effusion, ascites	30	stabilization of symptoms	N	SD	irThyreoiditis (grade 2)
4	male	33	cutaneous melanoma	Pembrolizumab	Pleural and pericardial effusion, ascites, swelling of face and scrotum	12	cessation of symptoms	N	PD	irThyreoiditis (grade 3), irThrombocytopenia (grade 4)

*NSCLC* Non-small cell lung cancer

## Discussion

This case series of patients with immune-induced polyserositis demonstrates that ICI can induce these potentially life-threatening side effects with cardiac and pulmonary affection. Additionally, we show that peripheral edema can occur. As shown, patients with irSerositis may benefit from corticosteroids although symptoms tend to recur.

While pericardial effusions in patients with advanced lung cancer after treatment with nivolumab have been reported [10, 18, 20] and might be life-threatening due to pericardial tamponade [12], no reports on irPolyserositis exist to our knowledge. The onset of irSerositis is often acute with respiratory failure and chest pain [13–16], but can also be slowly progressive with weight gain or progressive exertional dyspnea (Case 1–4 [7]). Symptom onset after initiation of immunotherapy ranged from 8 to 52 weeks but earlier and later onset ranging from seven days [17] to 72 weeks [18] or even as late as 200 weeks after start of checkpoint therapy [19] has been reported. Diagnosis may be difficult since progression of disease with malignant infiltrates has to be distinguished from immune-mediated effusions and can appear simultaneously. Furthermore, infectious causes have to be taken into account and excluded before initiating a therapy with corticosteroids. Histopathological findings are essential to differentiate between malignant effusion and an immune-related adverse event, where the latter show a dominance of lymphocytes and the absence of malignant cells. Importantly, the possibility of a combination of both should also be considered, as assumed in case 1. Naturally, in the cases with a dominance of lymphocytes in the pathological findings of the pleural fluid (case 2, 3 and 4) therapy with corticosteroids was more successful than in case 1, in which also malignant cells were detected in the pleural—as well as in the pericardial fluid.

Treatment of irSerositis represents a challenge: In the review by Saade et al. of patients with pericardial effusions, 69% required pericardiocentesis, immunotherapy was stopped in more than half of the patients and 19% of the patients experienced recurrent pericardial effusion. Only 44% of patients received corticosteroids and response to steroids was not reported [7]. In accordance with the case from Shaheen et al. [20] reporting beneficial steroid therapy, our patients all received steroids and benefited in the majority of cases. Although steroid treatment led to improvement of the effusions in two cases, multiple punctures were necessary in the other two patients and effusions recurred even after discontinuation of immunotherapy. In case of resistance to steroids, the initiation of a second-line immunosuppressive treatment with infliximab might be promising [18]. Interestingly, one case in the literature spontaneously resolved despite continuation of immunotherapy [9].

Our patients all developed other irAE in addition to the serositis while for the NSCLC patients other irAEs were documented in 44% of cases only [7]. The significance of irSerositis for tumor outcome is controversial. While Kolla and Patel reported a complete response in their patient, Epaillard et al. postulated a poorer prognosis for patients with pleural effusion in NSCLC [10]. We saw mixed outcomes of tumor therapy with stable disease in one case.

We are the first to report of irPolyserositis with involvement of the pericardium, pleura, peritoneum and peripheral edema. In conclusion, irSerositis might be more frequent than previously thought since especially in metastatic patients it is mostly assumed to be malignant. Our cases with repeated pathological investigations excluding malignant effusions however illustrate that irSerositis is an important differential diagnosis. If steroid-refractory, second-line therapy with infliximab might be useful.

## Data Availability

Data and material are available.

## Abbreviations

AJCC: American joint committee on cancer
bw: Body weight
CT: Computed tomography
CTCAE: Common terminology criteria of adverse events
fT4: Free thyroxine
HIT: Heparin induced thrombocytopenia
HU: Hounsfield units
ICI: Immune checkpoint inhibitors
MRI: Magnetic resonance imaging
NSCLC: Non-small cell lung cancer
PET/CT: Positron emission tomography/computed tomography
SERIO: Side effect registry immuno-oncology
